# Effect of different surface treatments on the bond strength of zirconia to resin cement. An *in vitro* study

**DOI:** 10.3389/fdmed.2026.1778379

**Published:** 2026-02-26

**Authors:** Aya El Feghaly, Elie Nasr, Monika Lukomska-Szymanska, Ewa Zmysłowska-Polakowska, Georges Najjar, Ryan Harouni, Rim Bourgi, Louis Hardan

**Affiliations:** 1 Resident at the Department of Aesthetic and Prosthetic Dentistry, Faculty of Dental Medicine, Saint Joseph University of Beirut, Beirut, Lebanon; 2 Department of Prosthodontic Dentistry, Faculty of Dental Medicine, Saint-Joseph University, Beirut, Lebanon; 3 Department of General Dentistry, Medical University of Lodz, Lodz, Poland; 4 Department of Endodontics, Medical University of Lodz, Łódź, Poland; 5 Department of Removable Dentistry, Faculty of Dental Medicine, Saint-Joseph University, Beirut, Lebanon; 6 Department of Restorative and Esthetic Dentistry, Faculty of Dental Medicine, Saint-Joseph University of Beirut, Beirut, Lebanon

**Keywords:** biomic LiSi connect, resin cement, shear bond strength, surface treatment, thermocycling, zirconia, Zircos-E

## Abstract

**Introduction:**

Achieving a durable and stable bond between zirconium dioxide (zirconia) and resin cement remains a significant clinical challenge. This study aimed to investigate the effects of two novel zirconia surface treatments on the shear bond strength (SBS) between zirconia and resin cement following thermocycling.

**Methods:**

Seventy-five CAD/CAM zirconia specimens (8 × 10 × 8 mm) were fabricated and randomly assigned to five groups (*n* = 15): control (no treatment); air particle abrasion (APA); Zircos-E etching; APA+Zircos-E; Biomic LiSi Connect. All specimens were bonded using a zirconia primer, adhesive system, and dual-cure resin cement, except for Biomic LiSi Connect, which did not receive the zirconia primer treatment. Subsequently, all specimens underwent thermo-cycling. SBS was measured, and failure modes were classified. Statistical analysis revealed significant differences in SBS among the groups (*p* < 0.001).

**Results and Discussion:**

Zircos-E etching, APA+Zircos-E and Biomic LiSi Connect exhibited significantly higher bond strength than the control, with Biomic LiSi Connect demonstrating the highest mean SBS (*p* < 0.001). Failure mode analysis showed no statistically significant differences among groups. Both Zircos-E and Biomic LiSi Connect significantly enhanced the bond strength.

## 1 Introduction

Reliable bonding to zirconia restorations remains a persistent clinical challenge in adhesive dentistry. Despite zirconia's widespread use for crowns, fixed dental prostheses, and implant-supported restorations, debonding failures continue to occur, particularly with conventional adhesive protocols. These complications affect restoration longevity and may require retreatment, increasing biological and financial burdens for clinicians and patients (1–4). The development of high-strength ceramics, including aluminum oxide (Al₂O₃) and zirconium dioxide (ZrO₂), has provided superior mechanical performance and long-term stability (5, 6). However, while the incorporation of ZrO₂ enhances mechanical strength, it simultaneously reduces the glassy matrix and silica content, making the material more resistant to acid attack (7). Although HF etching effectively enhances the surface roughness and wettability of silica-based ceramics (8), its impact on zirconia is minimal (9). Due to its chemically inert nature, zirconia therefore requires alternative surface modification strategies to achieve durable adhesion with resin cement.

Several surface treatment methods have been introduced to improve zirconia's bondability, including aluminium oxide (Al_2_O_3_) particle air abrasion, tribochemical silica coating, erbium-doped yttrium aluminium garnet (Er:YAG) laser conditioning, primer application, and silica nanofilm deposition. Among these, the use of 10-methacryloyloxydecyl dihydrogen phosphate (MDP)–containing agents has become the gold standard for zirconia surface treatment, as MDP forms a stable chemical bond with the zirconia surface (9). The combination of Al₂O₃ air abrasion followed by the application of MDP-containing primers or resin cements is currently regarded as the most reliable and effective bonding protocol in clinical practice (10). However, despite its widespread clinical acceptance, this protocol is not without limitations, particularly with respect to long-term bond durability under thermal and mechanical stresses encountered in the oral environment.

On the other hand, novel multi-acid etching solutions such as Zircos-E have been developed to modify zirconia surfaces by enhancing surface roughness (11). *In vitro* studies have provided valuable insights into the shear bond strength (SBS) and surface behaviour of zirconia treated with Zircos-E (6, 12). These emerging approaches aim to simplify clinical procedures while improving bonding predictability; however, their effectiveness relative to established protocols remains a subject of ongoing investigation.

Moreover, LiSi Connect (Aidite, Qinhuangdao, China) has been introduced as an innovative surface treatment system designed to address the limitations of conventional zirconia pretreatments (13, 14). It combines the mechanical resilience of zirconia with the chemical reactivity of silica-based ceramics, potentially improving micromechanical retention and chemical bonding (14, 15).

Thus, this *in vitro* study aimed to assess the impact of different surface treatments on adhesion between zirconia and resin cement after thermocycling. The null hypothesis stated that air particle abrasion, Zircos-E and Biomic LiSi Connect would have no significant effect on the SBS or failure mode of zirconia bonded to resin cement compared to conventional surface treatments after thermocycling.

## 2 Materials and methods

A total of 75 CAD/CAM zirconia specimens (8 × 10 × 8 mm) were prepared for this study, which was approved by the Ethical Committee of Saint-Joseph University of Beirut (Beirut, Lebanon; ref. USJ-2023-260). The sample size was determined by a one-way ANOVA power analysis using G*Power software (version 3.1.9.7; Heinrich Heine University, Düsseldorf, Germany), with a power of 0.9, an alpha level of 0.05, and an effect size of 0.55. This analysis indicated that a minimum of 75 specimens (15 per group) were required for five groups. Following the assigned treatments, all specimens were bonded using a zirconia primer, bonding agent, and dual-cure resin cement. To simulate oral aging, thermocycling was performed for 5,000 cycles between 5°C and 55°C. SBS was measured using a universal testing machine, and failure modes were examined under a stereomicroscope (Olympus SZ51, Japan) at 10× magnification.

### 2.1 Materials and equipment

The materials and instruments utilized in this study included zirconia discs Ceramill ZI D Shape (AmannGirrbach, Herrschaftswiesen/6842 Kobtach, Austria), bonding agents (3M™ Adper™ Single Bond 2, 3M ESPE, St Paul, MN, USA), Bisco zirconia primers (BISCO Z-Prime, Bisco, Schaumburg, IL, USA), resin cement (DMG PermaCem 2.0, Ridgefield Park, NJ, USA), zirconia etchant Zircos-E (Bio Den Co., Ltd., Seoul, South Korea), and zirconia primer Biomic LiSi Connect (Aidite, Qinhuangdao, China).

### 2.2 Zirconia specimen preparation

Using CAD/CAM milling machine, 75 zirconia cubes were milled out of 4 zirconia discs (AmannGirrbach Ceramill ZI D Shape). The design of each zirconia specimens (8 mm × 10 mm × 8 mm) was drawn and exported as Standard Triangulation Language (STL) file to CAD/CAM software. Each disc was mounted in the milling machine in dry mode. Following milling, each cube underwent a cleaning process using air jets, followed by air drying and placement in a ceramic oven for sintering in accordance with the manufacturer's instructions. The sintering process involved reaching a temperature of 1,550 °C with an increasing rate of 100 °C/min, maintaining this temperature for 2 h, and then decreasing the temperature at a rate of 100 °C/min. After that, the specimens were polished with 600 μm sandpaper under water-cooling conditions to achieve a flat surface. The specimens were cleaned in an ultrasonic bath with distilled water for 5 min, followed by drying with an air jet and finally assigned to their respective groups for surface treatment.

### 2.3 Surface treatment

The zirconia specimens were randomly assigned to five treatment groups. Control received no surface treatment. In group Air Particle Abrasion (APA), the zirconia samples were air-abraded using 110 µm aluminium oxide particles Cobra (Renfert GmbH, Hilzingen, Germany) for 20 s at a distance of 10 mm and a pressure of 2 bar with a sandblasting machine (Pudeng Enterprises Co., Ltd., Hsinchu, Taiwan). In group Zircos-E etching, the specimens were immersed in ZE for 30 min in an ultrasonic cleaner filled with water, operating at a frequency range of 20–60 kHz and a power of 0.2–1.0 W/cm^2^. After etching, the specimens were rinsed under cold running water for 2 min and then steamed for 30 s.

Group APA + Zircos-E involved a combination of air abrasion as described for APA followed by ZE etching. In group Biomic LiSi Connect, after milling and sintering, the specimens were cleaned using a steam machine, and the LiSi Connect coating was evenly sprayed 1–2 times from a distance of 10–15 cm on the bonding surface. The specimens were then sintered according to specific parameters: initial temperature 450 °C with a drying time of 1 min, heating rate of 80 °C/min, maximum temperature of 900 °C with a holding time of 1.5 min, and a vacuum rate of 100% at an opening temperature of 300 °C. The LiSi Connect layer crystallizes on the zirconia into a lithium-disilicate-type (Li_2_Si_2_O_5_) coating that is tightly integrated with the zirconia substrate.

After surface treatments, all zirconia specimens were cleaned, rinsed with water, and air-dried for 30 s. In all groups (except for Biomic LiSi Connect), zirconia primer (Z-Prime Plus, Bisco) was applied evenly with a microbrush for 60 s and gently air-dried to remove excess solvent. A thin layer of bonding agent (3M™ Adper™ Single Bond 2 Adhesive) was then applied over the primed surface, air-dried for 5 s, and light- cured using the Eighteeth curing pen (Eighteeth, Changzhou, China) for 20 s according to the manufacturer's instructions.

For LiSi Connect group, hydrofluoric acid gel 9.5% (Bisco) was applied to the LiSi Connect–treated surface for 60 s, thoroughly rinsed with water for 30 s, and air-dried for 30 s. Silane (Porcelain Primer, Bisco) was applied with a microbrush and allowed to set for 60 s, then the same bonding protocol was per-formed as performed in other groups.

A cylindrical polyethylene mould with an internal diameter of 2.38 mm and a height of 2.15 mm (Bonding Jig, Ultradent Products, Inc., South Jordan, UT, USA) was placed on the adhesive-coated zirconia surface. Dual-cure self-adhesive resin cement (PermaCem 2.0) was injected from the bottom to the top of the mould to prevent air bubble formation and then light-cured for 40 s.

### 2.4 Thermocycling

**Figure 1:**
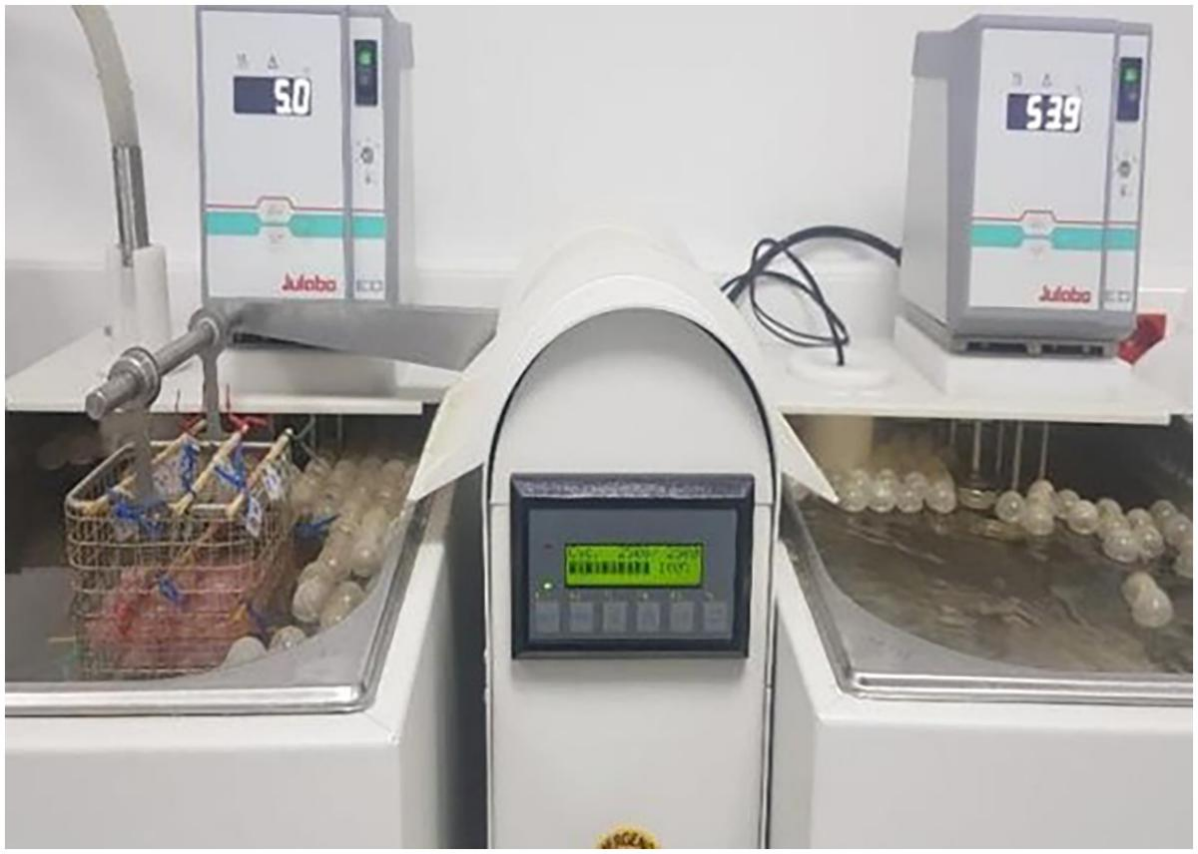
Specimens placed in the thermocycler, moving from the 5-degree tank to the 55-degrees.

The zirconia–resin assemblies were stored in distilled water at 37 °C for 24 h and subsequently subjected to thermocycling for 5,000 cycles between 5 °C and 55 °C to simulate six months of clinical service (Figure 1).

### 2.5 Shear bond strength and failure modes

**Figure 2:**
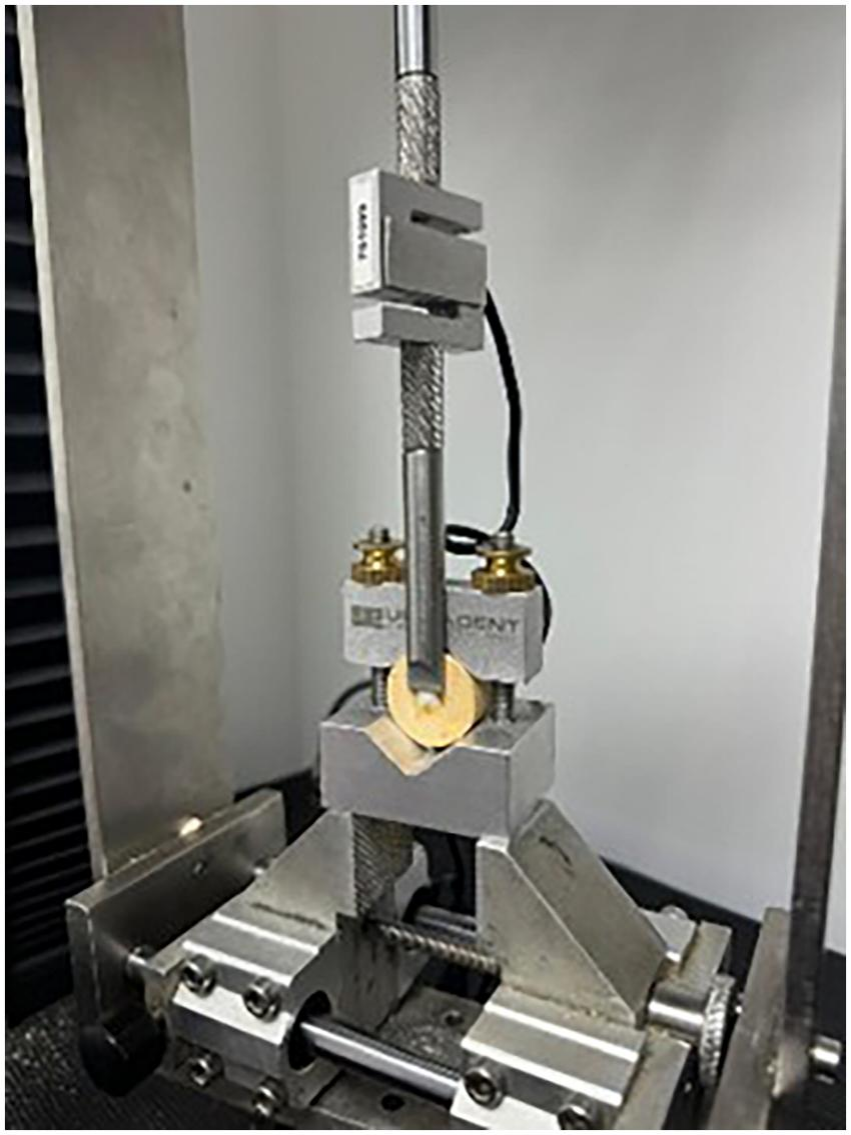
Universal testing machine measuring the shear strength of resin on a zirconia block.

SBS was measured using a universal testing machine (YLE GmbH Waldstrabe Bad Konig, Germany) at a crosshead speed of 1 mm/min until fracture (Figure 2) in accordance with ISO/TS 11405:2015. A blunt knife-edge blade was positioned perpendicularly, touching only the bonding interface: between the zirconia and the resin cement, aiming to assess the maximum shear bond strength at the failure point between zirconia and the resin cement.

The failure load of each specimen was measured in Newtons. The SBS was calculated as follows: SBS (MPa) = load (N)/area (mm2). The mode of fracture was then evaluated by inspecting the fractured surfaces under a stereo microscope (Olympus SZ51 Stereo Zoom Microscope, Japan) at 10× magnification and classified as adhesive, cohesive, or mixed failures (16):

**Adhesive:** Failure occurred at the interface between the ceramic surface and the bonding material, with no visible resin residue on the ceramic surface.
**Cohesive:** Failure occurred within the resin, leaving visible resin residue on the ceramic surface.
**Mixed:** Resin residue was visible on part of the ceramic surface, while other areas showed no residue.

### 2.6 Statistical analysis

Data analysis was performed using RStudio Version 2024.12.0. Normality was assessed using the Shapiro–Wilk test and homogeneity of variances using Levene's test. Differences in shear bond strength were analyzed using Welch's one-way ANOVA followed by the Games–Howell *post hoc* test. Failure mode distribution was evaluated using Fisher's Exact Test. Statistical significance was set at *p* ≤ 0.05.

## 3 Results

### 3.1 Bond strength comparison

The bond strength values for the five surface treatment groups were analysed using descriptive statistics, and a one-way ANOVA was initially performed to evaluate intergroup differences. The analysis revealed a significant difference in bond strength among the groups (Table 1). The primary objective was to assess the adhesive performance and durability of the various bonding protocols by measuring bond strength (MPa).

**Table 1:** Shear bond strength data for different surface treatment groups.

Group	Mean	Median	SD
Control	8.53	8	7.11
APA	29.3	25	23.8
Zircos-E Etching	34.9	30	20.3
APA + Zircos-E	53.4	48	31
LiSi Connect	101.0	98	15.6

The shear bond strength values of the five surface treatment groups are summarized in Table 1. showing that the LiSi Connect exhibited the highest mean bond strength, whereas control showed the lowest values.

As the assumption of homogeneity of variances was violated (*p* = 0.005), Welch's one-way ANOVA was used for intergroup comparison. This analysis revealed a statistically significant difference in bond strength among groups [*F* (4, 32.06) = 105.78, *p* < 0.001].

### 3.2 Pairwise comparisons

Given the significant result of Welch's one-way ANOVA, pairwise comparisons were performed using the Games–Howell *post hoc* test. (Table 2).

**Table 2:** Results of the pairwise comparisons.

Comparison	Difference	Lower bound	Upper bound	Adjusted *p*-value
APA vs. Control	20.73	−0.082	42.28	0.063
Zircos-E Etching vs. Control	26.33	4.78	47.88	0.011*
APA + Zircos-E vs. Control	44.87	23.32	66.42	<0.001
LiSi Connect vs. Control	92.20	78.90	105.40	<0.001*
Zircos-E Etching vs. Air Abrasion	5.6	−15.95	27.15	0.901
APA + Zircos-E vs. APA	24.13	2.58	45.68	0.022*
APA + Zircos-E vs. Zircos-E Etching	18.53	−3.02	40.08	0.116
LiSi Connect vs. APA	71.53	49.80	93.10	<0.001*
LiSi Connect vs. Zircos-E Etching	65.87	46.50	85.20	<0.001*
LiSi Connect vs. APA + Zircos-E	47.33	20.60	74.00	<0.001*

* The important values for this study showing significant results of *p* value.

Post hoc pairwise comparisons using the Games–Howell test indicate that, LiSi Connect exhibited significantly higher bond strength than all other groups, while Control demonstrated significantly lower bond strength compared to Zircos-E Etching, APA + Zircos-E, and LiSi Connect. APA did not differ significantly from Control, Zircos-E Etching, or APA + Zircos-E.

**Figure 3:**
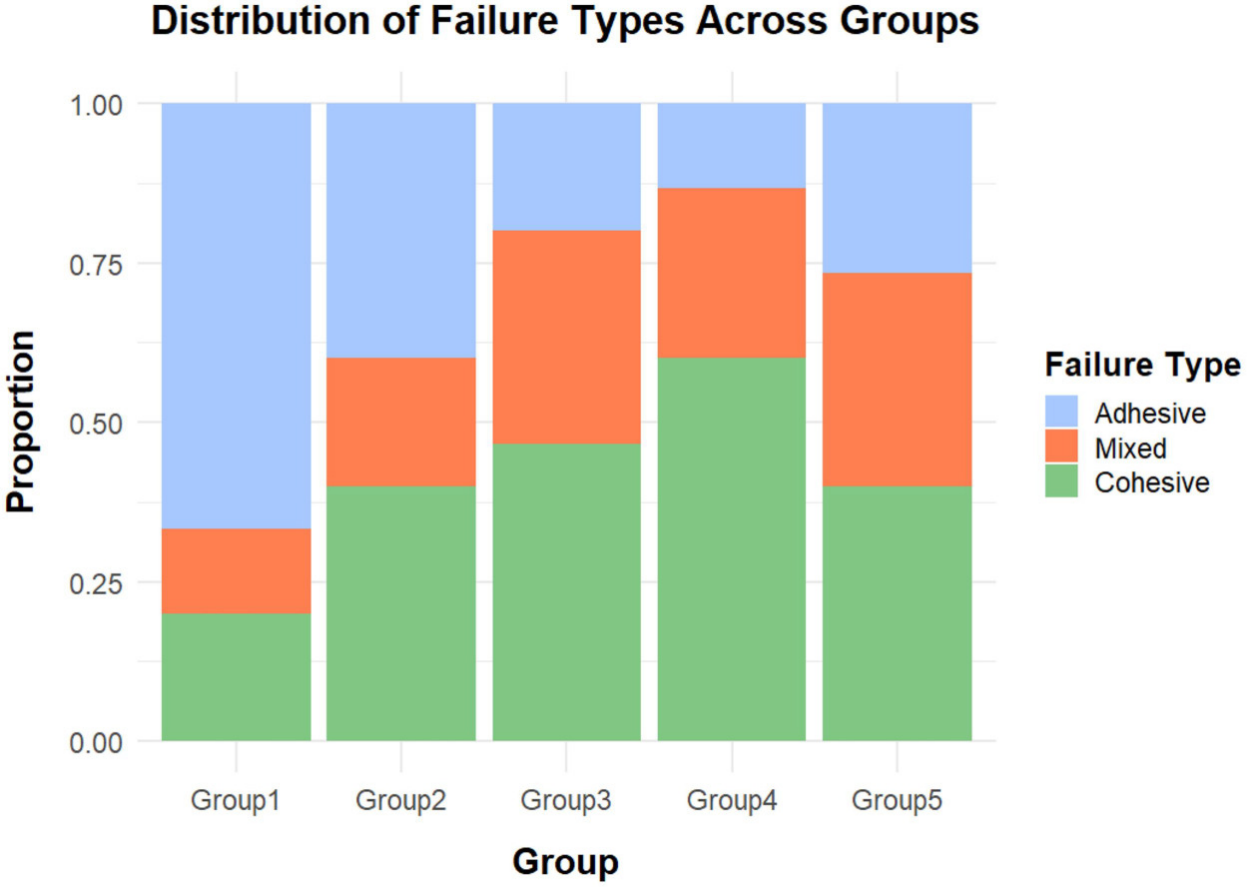
Stacked bar chart showing proportion of cohesive, adhesive and mixed failures.

Failure modes were categorized as adhesive, cohesive, or mixed. The distribution of failure types varied among the groups, with the control group predominantly exhibiting adhesive failures, whereas Zircos-E Etching and APA + Zircos-E showed higher proportions of cohesive and mixed failures (Figure 3). However, Fisher's Exact Test (*p* = 0.1472) indicated that no statistically significant association was observed between failure type and surface treatment.

## 4 Discussion

The longevity and clinical success of indirect restorations depend on the bond between the restoration and the tooth substrate influenced by material selection and protocols. Bonding to zirconia presents challenges due to its polycrystalline structure and lack of silica phase, rendering etching ineffective (16). Surface treatments such as air abrasion and acid etching with resin cements, are critical for zirconia retention. Although HF etching was considered ineffective for zirconia, recent evidence suggests that specific conditions can alter the surface morphology to improve bonding (17).

Based on the literature, sandblasting with alumina particles of varying sizes and pressures has been widely investigated to improve zirconia bonding (15, 18–21). Moderate-pressure air-borne particle abrasion (APA) has consistently been shown to enhance surface roughness and micromechanical retention while minimizing the risk of subsurface damage or tetragonal-to-monoclinic phase transformation (12, 22, 23). Consequently, the sandblasting parameters used in the present study were selected to represent a clinically “safe” and effective protocol that balances bonding efficacy with material integrity (12). While excessive air abrasion can induce subsurface damage or tetragonal-to-monoclinic phase transformation, the moderate pressure and particle size used in this study were selected based on protocols known to enhance bonding while minimizing such risks (12, 22).

Zircos-E is a multi-acid etching system developed for the chemical surface modification of zirconia ceramics. Unlike mechanical pretreatments, Zircos-E chemically activates the inert zirconia surface through acid reactions that generate micro-porosities and surface hydroxyl groups, increasing surface energy and reactivity (12). This hydroxylated surface promotes chemical interaction with phosphate-based functional monomers, such as 10-MDP, facilitating stable Zr–O–P bonding while preserving the crystalline integrity of zirconia.

The present study evaluated SBS and failure modes among five experimental groups. For the Zircos-E treatment, etching significantly improved bond strength compared to air abrasion and untreated controls. This increase is likely due to surface modifications at the grain boundaries, creating micro-porosities that enhance mechanical retention. Moreover, specimens subjected to combined etching and APA exhibited higher SBS than control, APA or Zircos-E etching. This observation is supported by literature (19, 24). The combined mechanical and chemical treatments provide a compounded effect: air abrasion improves micromechanical retention, while chemical etching enhances surface reactivity, promoting superior adhesion (25). Although APA effectively increases surface roughness and facilitates micromechanical retention, it provides limited chemical activation of the zirconia surface, which may explain its comparatively lower bonding effectiveness when used alone (18, 26). Chemical etching with Zircos-E further enhances bonding by creating micro-porosities of varying morphologies and increasing the effective bonding area, possibly resulting in a more stable resin–zirconia interface (12, 22, 23).

On the other hand, LiSi Connect is a pretreatment system intended for the surface modification of zirconia ceramics. The ultra-thin coating (∼6–10 µm) is designed to preserve the original geometry of the restoration while converting the inert zirconia surface into a substrate with a glass-ceramic-like bonding behaviour and, will penetrates slightly into the zirconia surface and crystallises it, producing a lithium disilicate micro-coating embedded on the zirconia (15). It highlights the additive benefits of mechanical and chemical surface modifications and the efficacy of the novel LiSi Connect approach. LiSi Connect method enables conventional adhesive protocols without altering zirconia's intrinsic physical properties. Hydrofluoric acid etching subsequently generates hydroxyl groups (-OH) on the surface, improving resin cement affinity and chemical bonding (27). The superior bonding performance observed with LiSi Connect is in agreement with previous studies reporting that lithium-silicate coatings on zirconia produce bond strengths comparable to those achieved with lithium disilicate ceramics (12, 27, 28). From a clinical perspective, this strategy offers a promising means of improving bonding predictability and durability without compromising zirconia's intrinsic mechanical properties.

Failure mode analysis revealed a shift from predominantly adhesive failures in untreated specimens toward mixed and cohesive failures in chemically treated groups, suggesting improved interfacial integrity. Although no statistically significant association between failure mode and surface treatment was detected, the observed trends align with existing literature indicating that chemical surface conditioning enhances zirconia–resin adhesion (28, 29). These observations support prior findings, highlighting those mechanical treatments alone may not achieve optimal zirconia bonding (28). Whereas chemical surface conditioning significantly enhances resin–zirconia adhesion, potentially approaching the bond strength observed in enamel (20, 30). Further investigations encompassing a broader range of materials and clinical conditions are warranted to optimize zirconia bonding strategies.

To conclude, based on the current literature and the findings of this study, advanced chemical conditioning systems such as Zircos-E and LiSi Connect demonstrated superior results. The significant increase in SBS obtained with LiSi Connect, even after thermocycling equivalent to six months of clinical aging, confirms its potential for long-term durability.

Several limitations of this study should be acknowledged. Although thermocycling was applied to simulate thermal aging, the *in vitro* design cannot fully reproduce the complex intraoral environment, where restorations are subjected to cyclic mechanical loading, moisture, enzymatic activity, and pH fluctuations. In addition, only one generation of zirconia was evaluated, which limits the generalizability of the findings to other zirconia formulations that may respond differently to surface treatments. Furthermore, both Zircos-E and LiSi Connect are technique-sensitive procedures that require strict control of application parameters and appropriate safety measures, potentially affecting their reproducibility in clinical practice. Importantly, any claims regarding clinical equivalence or long-term durability should be interpreted with caution, as the present findings are limited to short-term *in vitro* performance, and long-term bonding stability remains to be demonstrated. Therefore, future studies should include different generations of zirconia, extended aging protocols, and well-designed clinical trials to validate the long-term durability, safety, and clinical applicability of these surface treatment strategies.

## 5 Conclusions

Within the constraints of the present study, it can be concluded that:

1. Surface treatment protocols exert a significant influence on shear bond strength (SBS).
2. The combination of Zircos-E etching with air abrasion results in a synergistic enhancement of SBS.
3. Among the surface treatments evaluated, the LiSi Connect system achieves the highest SBS values. Therefore, LiSi Connect demonstrates promising results for augmenting zirconia bonding through the integration of mechanical and chemical modifications, though further long-term clinical validation is needed.

## Data Availability

The raw data supporting the conclusions of this article will be made available by the authors, without undue reservation.

## References

[1] Tzanakakis EG Tzoutzas IG Koidis PT. Is there a potential for durable adhesion to zirconia restorations? A systematic review. J Prosthet Dent. (2016) 115(1):9–19. 10.1016/j.prosdent.2015.09.008 26548872

[2] Quigley NP Loo DS Choy C Ha WN. Clinical efficacy of methods for bonding to zirconia: a systematic review. J Prosthet Dent. (2021) 125(2):231–40. 10.1016/j.prosdent.2019.12.017 32115220

[3] Fabbri G Ban G Pulcini C Cerutti A Özcan M. Clinical performance of complete-arch implant-supported rehabilitations using monolithic lithium disilicate restorations bonded to CAD/CAM titanium and zirconia frameworks up to 5 years. Eur J Prosthodontics Restorative Dentistry. (2022) 30(4):296–304. 10.1922/EJPRD_2069Fabbri09 35438262

[4] Shahmiri R Standard OC Hart JN Sorrell CC. Optical properties of zirconia ceramics for esthetic dental resto-rations: a systematic review. J Prosthet Dent. (2018) 119(1):36–46. 10.1016/j.prosdent.2017.07.009 28927925

[5] Chaiyabutr Y McGowan S Phillips KM Kois JC Giordano RA. The effect of hydrofluoric acid surface treat-ment and bond strength of a zirconia veneering ceramic. J Prosthet Dent. (2008) 100(3):194–202. 10.1016/S0022-3913(08)60178-X 18762031

[6] Qi B Liang S Li Y Zhou C Yu H Li J. Zro2 matrix toughened ceramic material-strength and toughness. Adv Eng Mater. (2022) 24(6):2101278. 10.1002/adem.202101278

[7] Smielak B Klimek L. Effect of hydrofluoric acid concentration and etching duration on select surface roughness parameters for zirconia. J Prosthet Dent. (2015) 113(6):596–602. 10.1016/j.prosdent.2015.01.001 25799283

[8] Sales A Rodrigues SJ Mahesh M Ginjupalli K Shetty T Pai UY Effect of different surface treatments on the micro-shear bond strength and surface characteristics of zirconia: an *in vitro* study. Int J Dent. (2022) 2022(1):1546802. 10.1155/2022/1546802 35464102 PMC9023206

[9] Sadid-Zadeh R Strazzella A Li R Makwoka S. Effect of zirconia etching solution on the shear bond strength between zirconia and resin cement. J Prosthet Dent. (2021) 126(5):693–7. 10.1016/j.prosdent.2020.09.016 33162113

[10] Yue X Hou X Gao J Bao P Shen J. Effects of MDP-based primers on shear bond strength between resin cement and zirconia. Exp Ther Med. (2019) 17(5):3564–72. 10.3892/etm.2019.7382 30988738 PMC6447804

[11] Cho J Kim SJ Shim JS Lee KW. Effect of zirconia surface treatment using nitric acid- hydrofluoric acid on the shear bond strengths of resin cements. J Adv Prosthodont. (2017) 9(2):77–84. 10.4047/jap.2017.9.2.77 28435615 PMC5397592

[12] Conner C Andretti F Hernandez AI Rojas-Rueda S Azpiazu-Flores FX Morrow BR Surface evaluation of a novel acid-etching solution for zirconia and lithium disilicate. Materials (Basel). (2025) 18(12):2912. 10.3390/ma18122912 40573041 PMC12195062

[13] Shen D Wang H Shi Y Su Z Hannig M Fu B. The effect of surface treatments on zirconia bond strength and durability. J Funct Biomater. (2023) 14(2):89. 10.3390/jfb14020089 36826888 PMC9968119

[14] Chen Z Zhou Y Li D Zhang M Zhou B Hao P. Does the internal surface treatment technique for enhanced bonding affect the color, transparency, and surface roughness of ultra-transparent zirconia? Clin Oral Investig. (2024) 28(9):473. 10.1007/s00784-024-05847-4 39110133

[15] Wu SH Lai SY Lee IT Mine Y Huang HY Peng TY. Handheld nonthermal plasma augmentation of glass–ceramic spray deposition on zirconia surface characterization and MG-63/HGF-1 cell behavior: an *in vitro* study. J Funct Biomater. (2025) 16(11):421. 10.3390/jfb16110421 41295076 PMC12653843

[16] Priyavardhini P. Assessment of different surface treatments and bonding agents on the shear bond strength of aged composite-an in vitro study (Master’s thesis). Rajiv Gandhi University of Health Sciences, India.

[17] Ria A Ibrahim H. Adhesion to zirconia: factors affect bonding to zirconia. ERU Res J. (2025) 4(1):2190–209. 10.21608/erurj.2025.318931.1181

[18] Moon JE Kim SH Lee JB Han JS Yeo IS Ha SR. Effects of airborne-particle abrasion protocol choice on the surface characteristics of monolithic zirconia materials and the shear bond strength of resin cement. Ceram Int. (2016) 42(1):1552–62. 10.1016/j.ceramint.2015.09.104

[19] Su N Li Y Yunmao L Wenjia L Hai Z Xin L The effect of various sandblasting conditions on surface changes of dental zirconia and shear bond strenght between zirconia core and indirect composite resin. J Adv Prosthodont. (2015) 7(3):214–23. 10.4047/jap.2015.7.3.214 26140173 PMC4486617

[20] Scaminaci Russo D Cinelli F Sarti C Giachetti L. Adhesion to zirconia: a systematic review of current condi-tioning methods and bonding materials. Dentistry Journal. (2019) 7(3):74. 10.3390/dj7030074 31374820 PMC6784479

[21] Zhang Q Yao C Yuan C Zhang H Liu L Zhang Y Evaluation of surface properties and shear bond strength of zirconia substructure after sandblasting and acid etching. Mater Res Express. (2020) 7(9):095403. 10.1088/2053-1591/abb5c9

[22] Kui A Buduru S Labuneț A Sava S Pop D Bara I Air particle abrasion in dentistry: an over-view of effects on dentin adhesion and bond strength. Dentistry Journal. (2024) 13(1):16. 10.3390/dj13010016 39851592 PMC11764507

[23] Lee JH Kim SH Han JS Yeo IL Yoon HI. Optical and surface properties of monolithic zirconia after sim-ulated tooth-brushing. Materials (Basel). (2019) 10:1158. 10.3390/ma12071158 PMC6480371 30974750

[24] Pilo R Dimitriadi M Palaghia A Eliades G. Effect of tribochemical treatments and silane reactivity on resin bonding to zirconia. Dent Mater. (2018) 34(2):306–16. 10.1016/j.dental.2017.11.006 29183673

[25] Kim M-J Kim YK Kim K-H Kwon T-Y. Shear bond strengths of various luting cements to zirconia ceramic: surface chemical aspects. J Dent. (2011) 39(11):795–803. 10.1016/j.jdent.2011.08.012 21907260

[26] Al-Amari AS Saleh MS Albadah AA Almousa AA Mahjoub WK Al-Otaibi RM A comprehensive review of techniques for enhancing zirconia bond strength: current approaches and emerging innovations. Cureus. (2024) 16(10). 10.7759/cureus.70893 39497891 PMC11534439

[27] Veríssimo AH Moura DM Tribst JP Araújo AM Leite FP. Effect of hydrofluoric acid concentration and etching time on resin-bond strength to different glass ceramics. Braz Oral Res. (2019) 33:e041. 10.1590/1807-3107bor-2019.vol33.0041 31508723

[28] Peng T-Y Kang C-M Feng S-W Hung C-Y Iwaguro S Dan-Jae Lin D-J. Effects of glass-ceramic spray deposition manipulation on the surface characteristics of zirconia dental restorations. Ceram Int. (2022) 48(20):29873–81. 10.1016/j.ceramint.2022.06.252

[29] Kim SH Choi YS Kang KH Att W. Effects of thermal and mechanical cycling on the mechanical strength and surface properties of dental CAD-CAM restorative materials. J Prosthet Dent. (2022) 128(1):79–88. 10.1016/j.prosdent.2020.11.014 33546857

[30] Aschheim KW. Esthetic Dentistry: A Clinical Approach to Techniques and Materials. St. Louis (MO): Elsevier Health Sciences (2014).

